# Prevention of Transfusion-Transmitted Malaria and Chagas Disease in Non-Endemic Countries: An 8-Year Study of Seroprevalence Among Donors at Risk in Tuscany (Central Italy)

**DOI:** 10.3390/pathogens15010020

**Published:** 2025-12-23

**Authors:** Valentina D. Mangano, Barbara Pinto, Roberto Marotta, Luca Galli, Giovanna Antonella Moscato, Antonella Lupetti, Fabrizio Bruschi

**Affiliations:** 1 Dipartimento Ricerca Traslazionale e Nuove Tecnologie in Medicina e Chirurgia, Università di Pisa, Via Savi 10, 56126 Pisa, Italy; barbara.pinto@unipi.it (B.P.); antonella.lupetti@unipi.it (A.L.); 2 Dipartimento Professioni Tecnico Sanitarie, Azienda Ospedaliero-Universitaria Pisana, Via Paradisa 2, 56124 Pisa, Italy; r.marotta@ao-pisa.toscana.it; 3 Servizio di Medicina Trasfusionale, Ospedale di Lucca, Via Guglielmo Lippi Francesconi 556, 55100 Lucca, Italy; luca.galli@uslnordovest.toscana.it; 4 UO Laboratorio di Analisi chimico cliniche, Azienda Ospedaliero-Universitaria Pisana, Via Paradisa 2, 56124 Pisa, Italy; g.moscato@ao-pisa.toscana.it; 5 SOD di Microbiologia Micologica, Azienda Ospedaliero-Universitaria Pisana, Via Paradisa 2, 56124 Pisa, Italy; 6 Programma Monitoraggio delle Parassitosi e Formulazione Algoritmi Diagnostici, Azienda Ospedaliero-Universitaria Pisana, Via Paradisa 2, 56124 Pisa, Italy

**Keywords:** *Trypanosoma cruzi*, *Plasmodium*, Chagas disease, Malaria, blood, transfusion, screening, prevention

## Abstract

Vector-borne parasites might be transmitted through transfusion, notably *Plasmodium* spp. and *Trypanosoma cruzi*. Prevention strategies include blood donor screening, deferral, and blood unit treatment by pathogen inactivation methods. At the end of 2015, in line with European guidelines, Italian legislation introduced a questionnaire to identify donors at risk and their screening by serological methods. In early 2016, the Laboratory of Parasitology at Pisa University Hospital started the serological analysis of donors at risk, referring to Transfusion Services located in northwestern Tuscany. The aim of the present study was to describe the prevalence of seropositive donors observed during 8 years of screening. Donors at risk of transmitting malaria were screened by ELISA (Enzyme Linked Immunosorbent Assay). The DRG ELISA kit was employed until 2020, when it was substituted by the Euroimmun ELISA kit based on the results of a comparative evaluation of available commercial kits. Seropositive donors were offered the possibility of *Plasmodium* DNA testing by Loop-Mediated AMPlification (LAMP) to exclude current infection. Donors at risk of transmitting Chagas disease were screened by ICT employing recombinant antigen until 2021, when it was substituted by ELISA employing lysate antigen because of its higher accuracy. Seropositive donors were further tested by CLIA, and WB was performed in case of discordant results, according to WHO guidelines for diagnosis of chronic Chagas disease. A total of 3754 donors were tested for anti-*Plasmodium* antibodies, revealing a 6.8% (95% CI = 6.1–7.7%) seroprevalence. Seropositivity was higher among donors from Sub-Saharan Africa (42.9%; 95% CI = 36.1–49.9%) and Southeast Asia (10.6%; 95% CI = 6.7–16.4%). A lower seropositivity was observed when employing Euroimmun ELISA (4.8; 95% CI = 3.8–5.9%) than DRG ELISA (8.2%; 95% CI = 7.1–9.3%). Seropositivity dropped to 3.6% (95% CI = 2.4–5.6) in 2020, likely because of travel restrictions during the COVID-19 pandemic. None of the tested seropositive donors (n = 20) tested positive for *Plasmodium* DNA LAMP testing. A high proportion of seroreversion was observed after one year of testing. Among 4285 donors tested for anti-*T. cruzi* antibodies seroprevalence was 0.7% (95% CI = 0.5–1.1%), a higher value than what was observed in a recent national survey. All seropositive donors were born in Europe or Latin America. Seropositivity was apparently lower with ELISA (0.5%, 95% CI = 0.2–1.2%) than ICT (0.8%, 95% CI = 0.6–1.2%), possibly due to ELISA’s higher specificity, although the difference is not significant. No confirmed cases of chronic Chagas disease were identified. The study emphasizes the importance of defining the serological test employed for screening and the need to confirm seropositive results with further testing. The high seroreversion observed in the study suggests repeating seropositive donor screening after a year to minimize deferral and blood unit loss.

## 1. Introduction

Several vector-borne parasites can also be transmitted through blood transfusion, and the risk is particularly high for *Plasmodium* species causing malaria and for *Trypanosoma cruzi*, the etiological agent of Chagas disease (CD). Indeed, both parasites can survive under standard storage conditions (>2 weeks at 4–22 °C) used for whole blood and blood components [1,2]. Also, both parasites can cause asymptomatic infections and can therefore be carried by healthy donors.

Malaria is a life-threatening disease posing a major threat to global public health and is endemic in 83 countries. Over 250 million clinical cases and 600,000 related deaths were reported in 2023 [3]. In non-endemic countries, the large majority of cases are imported, i.e., infection is acquired in endemic areas by travellers, often to visit friends and relatives in the country of origin, while only a small minority are acquired locally due to transfusion or contaminated devices, transmission by an infected mosquito transported from endemic areas, or transmission by indigenous mosquitoes feeding on imported cases. In Europe, about 6000 cases are reported every year, with France, Germany, Spain, and Italy showing the highest numbers [4]. Individuals who have developed semi-immunity through repeated exposure to infectious mosquito bites in endemic areas may, as adults, carry low-density parasitemia—which can be persistent in the case of *Plasmodium falciparum* or chronic in the case of *Plasmodium malariae*—often without any clinical symptoms [5,6]. Moreover, *Plasmodium vivax* and *Plasmodium ovale* have dormant liver stages, called hypnozoites, which can remain in this state for months or even years before triggering a new blood-stage infection (relapse; [7]). Transfusion-transmitted malaria (TTM) is a relatively rare but very serious event; one hundred cases of TTM in non-endemic areas were documented up to 2019, and about half of these resulted in the death of the recipient [8].

Between 7 and 8 million people are estimated to be infected with *T. cruzi* worldwide, and CD causes nearly 10,000 deaths every year [9]. About 80% of cases are reported in Latin America, where the vector is endemic. The remaining 20% of cases are reported in the United States of America (300,000 cases) and Europe (80,000 cases), where CD represents an emerging public health problem, as well as in Canada, Australia, and Japan [10]. Spain is the European country with the highest number of estimated cases (up to 76,000 cases), followed by Italy (estimated up to 10,000 cases), France, and Germany (about 2000–3000 estimated cases in each) [11,12]. Following an acute phase, *T. cruzi* infection typically evolves into a chronic phase, marked by a low and intermittent parasitemia, which is asymptomatic in more than 70% of cases (indeterminate chronic phase). Blood transfusion, therefore, represents an important route of transmission for *T. cruzi* [13], and it has been estimated that up to 800 cases of transfusion-transmitted CD (TTCD) have occurred in recent decades globally [14,15]. In Europe, cases of TTCD have been reported in Spain [16,17]. In non-endemic areas, transmission through blood transfusion is the most frequent route of infection, along with solid organ transplantation and transplacental transmission [17].

The most common strategy to prevent pathogen transmission through blood transfusion involves identifying prospective donors at risk of infection and subsequently either deferring them for a defined period—a highly conservative approach—or performing laboratory testing on blood samples to detect the presence of infection biomarkers. An alternative prevention method is the treatment of blood units with pathogen reduction or inactivation methods, such as photochemical treatment [18]. For example, the Mirasol system, utilizing riboflavin (vitamin B2) and ultraviolet light, has shown promising efficacy in reducing *P. falciparum* (Log reduction ≥ 6.4) and *T. cruzi* (Log reduction ≥ 3.5) [19].

Currently, the World Health Organization (WHO) recommends that, to prevent transfusion-transmitted infections (TTI) and ensure blood safety, all blood donations should be tested for HIV, hepatitis B, hepatitis C, and syphilis, while screening for malaria and CD should be carried out based on risk exposure [20].

Guidelines from the European Directorate for the Quality of Medicine (EDQM) [21] recommend that candidate donors at risk of infection with *Plasmodium* spp. or *T. cruzi* should be admitted to donation only in the presence of a negative test for specific antibodies; a negative molecular test for *Plasmodium* DNA is also considered valid for TTM prevention.

There is wide variation between EU member states regarding the implementation of EDQM guidelines, with heterogeneous policies, but all rely on serology [22]. The use of serological tests, which are limited by the inability to distinguish between a past infection and an ongoing infection, is precautionary, since direct tests with sufficient sensitivity to detect the low parasitic densities characteristic of asymptomatic carriers—which are nonetheless sufficient for transfusion transmission—are not yet available. For example, as few as 10 red blood cells infected with *Plasmodium* spp. can cause TTM [1,8,23], a quantity corresponding to a parasite density below the detection limit of direct methods, including molecular assays, such as Polymerase Chain Reaction (PCR) or Loop-Mediated Isothermal Amplification (LAMP) [24].

In Italy, the current national regulation [25] introduced the use of a questionnaire to collect information about travel and residence in endemic areas and a mandatory serological screening for anti-*Plasmodium* and anti-*T. cruzi* antibodies in at-risk donors. According to this regulation, seropositive individuals are excluded from blood donation, subject to a 3-year deferral period for malaria and a permanent deferral for CD. However, the legislation does not specify which serological tests should be used for screening, nor the levels of specificity and sensitivity of such tests.

The Laboratory of Parasitology at Pisa University Hospital has been responsible since early 2016 for the serological analyses of at-risk donors referring to Transfusion Services located in northwestern Tuscany. The aim of the present study was to describe the prevalence and the characteristics of seropositive donors observed during 8 years of screening.

## 2. Materials and Methods

### 2.1. Study Population

The study included blood donors affiliated with the transfusion centres of the Provinces of Livorno, Lucca, Massa-Carrara, and Pisa (northwest area of the Tuscany Regional Health System) from February 2016 to February 2024, who were identified as at risk of infection with *Plasmodium* and/or *T. cruzi* through the questionnaire designed by the National Blood Centre. The questionnaire collects information on the country of birth, on countries of travel and/or residence, on birth from parents of Latin American origin, and on malaria or undiagnosed fever experienced during or until 6 months after a visit to an endemic country.

### 2.2. Ethics Considerations

Study subjects were recruited as part of the routine activities of the national screening program of candidate blood donors and gave written informed consent to participation [26]. The study was conducted according to Good Clinical and Laboratory Practices. The present report includes aggregated data only, and individual participants cannot be identified.

### 2.3. Laboratory Methods

For the prevention of transfusion-transmitted malaria, serum samples were tested for the presence of *Plasmodium*-specific antibodies by Enzyme-Linked ImmunoSorbent Assay (ELISA). The commercial kit “Malaria Ab” (DRG Diagnostics, Marburg, Germany), which detects IgM and IgG against *P. falciparum* and *P. vivax* recombinant antigens, was used in the period February 2016-March 2020, while the commercial kit “Anti-*Plasmodium* ELISA (IgG)” (Euroimmun, Padova, Italy), which detects IgG against *P. falciparum*, *P. vivax*, *P. malariae*, *P. ovale*, and *P. knowlesi* recombinant antigens, was used in the period April 2020-March 2024. The ELISA kit was changed because the repeatability of EuroImmun outperformed that of DRG in a comparative evaluation [27]. Candidate donors with a positive ELISA result were informed of the possibility of performing direct diagnosis on whole blood by Loop-Mediated AMPlification (LAMP) method using the commercial kit “Alethia Malaria” (Meridian Bioscience, Italy) to exclude current infection with *Plasmodium* spp. LAMP testing was conducted for all seropositive donors who requested it. All tests were performed according to manufacturers’ instructions.

For the prevention of transfusion-transmitted CD, sera samples were tested for the presence of *T. cruzi*-specific antibodies by ImmunoChromatographic Test (ICT) using the commercial kit “Chagas Quick Test” (Cypress Diagnostics, Hulshout, Belgium), which detects total antibodies against a recombinant antigen, in the period February 2016-February 2021, and by ELISA using the commercial kit “Chagatest ELISA lisado” (Weinerlab, Rosario, Argentina), which detects IgG against a parasite lysate antigen, in the period March 2021-March 2024. The test for screening was changed because ICT showed insufficient sensitivity in non-endemic countries [17] and was outperformed in a comparative evaluation of diagnostic accuracy for CD [28]. For the diagnosis of CD among seropositive donors, the ChemiLuminescent ImmunoAssay (CLIA) commercial kit “Architect Chagas” (Abbott, Italy), which detects IgG against recombinant antigens FP3, FP6, FP10, and TcF, was performed as a second antibody test. Since 2021, the Western blot (WB) commercial kit “Chagas Western blot IgG” (LDBIO Diagnostics, France), which detects IgG against parasite lysate antigen, has been performed as a confirmatory test in case of discordance between the first and second tests, in agreement with WHO guidelines [29]. All tests were performed according to manufacturers’ instructions.

### 2.4. Statistical Analysis

The count and frequency of seropositive candidate donors who were thereby excluded from donation were computed. Seroprevalence of anti-*Plasmodium* and anti-*T. cruzi* antibodies were compared according to sex/gender, age, and WHO region of birth of candidate donors, as well as according to year and assay of testing by likelihood ratio test. In the case of multiple antibody testing of the same donor with the same serological assay, results of tests performed at different times (1 month, 1 year, 2 years, 3 years, and more than 3 years) were compared. Results of multiple tests performed with different serological assays were not compared. A *p*-value ≤ 0.05 was considered significant. Statistical analysis was performed in STATAv.10.

## 3. Results

### 3.1. Malaria

From February 2016 to February 2024, a total of 4410 ELISA tests were performed to assess the presence of anti-*Plasmodium* antibodies among at-risk candidate blood donors. Of those tests, 175 (4%, 95% CI = 3.4–4.5) yielded inconclusive results, with OD values falling within the indeterminate range indicated by the ELISA kit manufacturer. The frequency of inconclusive results varied according to the ELISA test, and it was higher (OR = 2.3, 95% CI = 1.6–3.3, *p*-value < 0.001) when using the DRG kit (5.1%, 95% CI = 4.3–6.0) than when using the EUR kit (2.3%, 95% CI = 1.6–2.9).

The remaining 4235 tests with a conclusive result (positive or negative) were performed on 3754 donors and included multiple testing for a subset of donors. To analyze seropositivity (Table 1), only the first test result was taken into consideration. A positive result was observed for 238 subjects who were thereby excluded from donation, with a resulting seropositivity of 6.3% (95% CI = 6.1–7.7).

No differences in seropositivity were observed between female and male donors or according to the age group of donors. On the contrary, seropositivity varied greatly with the geographical origin of the donor (OR = 1.4, 95% CI = 1.3–1.5, *p*-value < 0.001): the highest seropositivity was observed for donors from the WHO African region, followed by donors from the WHO South East Asia, American, and West Pacific regions, while donors from the WHO European and Eastern Mediterranean regions showed the lowest seropositivity.

Also, seropositivity varied with the year of donation and testing (OR = 0.9, 95% CI = 0.9–1.0, *p*-value = 0.022): seropositivity decreased sharply in 2020, to resume previous levels in 2021, and decreased gradually from 2022. Finally, seropositivity varied with the ELISA used for testing (OR = 0.5, 95% CI = 0.4–0.7, *p*-value < 0.001), as it was lower for the EUR kit than for the DRG kit.

All seropositive donors were offered the possibility to perform a LAMP assay to detect *Plasmodium* DNA in peripheral blood in order to exclude a current malaria infection. LAMP tests were performed for 20 seropositive donors, all (100%) yielding a negative result.

**Figure 1:**
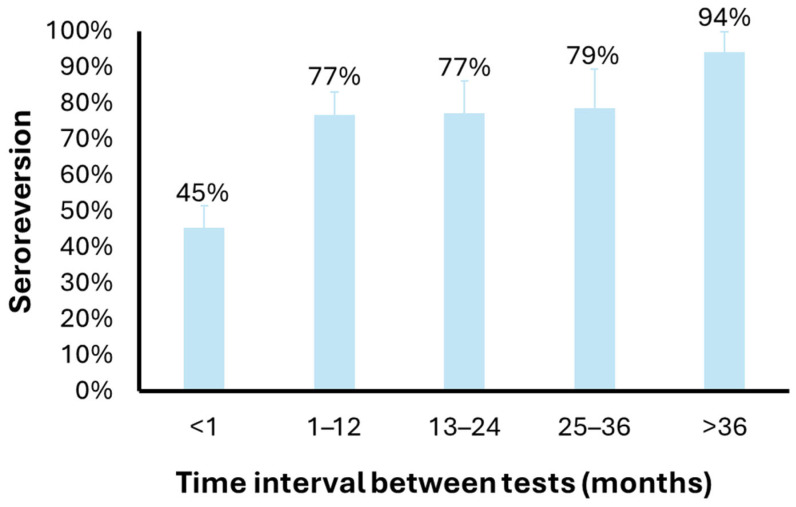
Anti-*Plasmodium* IgG seroreversion according to the time interval between tests. The bar plot shows the proportion of donors with a first result and a second negative result for anti-*Plasmodium* IgG testing, according to the time interval between the two tests.

A subset of seropositive subjects (N = 160) repeated the test after some time during the study period. Overall, 106 (66%) subjects who tested seropositive at the first ELISA test had a negative result at the second ELISA test. By stratifying the proportion of subjects with discordant test results by the time interval between the two tests, it is possible to observe an increase with time, from 45% for tests performed within a month (n = 64) to 77% for tests performed within 1 (n = 43) or 2 (n = 22) years, to 79% for tests performed within 3 years (n = 14) and 94% for tests performed after 3 years (n = 17) (Figure 1).

### 3.2. Chagas Disease

In the same time period, a total of 4285 ICT/ELISA tests were performed to assess the presence of anti-*T. cruzi* antibodies among an equal number of at-risk candidate blood donors. Thirty-two subjects had a positive test result and were therefore excluded from donation, with a resulting seroprevalence of 0.7% (Table 2). Among seropositive subjects, 28 were born in the WHO European region (Italy) and 4 in the WHO American region (Brazil, Ecuador). Seropositivity did not vary according to donor characteristics, testing method, or year of testing. None of the seropositive subjects was diagnosed with CD according to CLIA and WB test results.

## 4. Discussion

Malaria seropositivity was 6.8% in the overall donor population during the study period. To our knowledge, no national survey assessing the seroprevalence of *Plasmodium*-specific antibodies in blood donors has been conducted to date in Italy, and our findings therefore provide the first available seroprevalence estimate for an Italian region. Serological results for malaria showed a lower proportion of indeterminate results with the Euroimmun ELISA (2.3%) compared to the DRG ELISA (5.1%), a feature suggesting a higher precision of the former kit, as previously reported [27]. Notably, overall seropositivity also varied according to the assay used (Euroimmun: 5.9%; DRG: 9.3%). Unlike chronic CD, serology is not used for malaria diagnosis, which has limited research and development of in vitro diagnostic (IVD) tests. Nonetheless, the identification of antibody responses associated with recent *Plasmodium* exposure—currently explored for surveillance purposes in endemic countries [30]—could stimulate the development of methods suitable for blood donor screening in non-endemic settings. However, at present, only a few commercial assays are available, most of which rely on similar antigenic targets, and none has been approved for screening by the FDA [31].

A previous comparative evaluation of commercial ELISA kits demonstrated variable concordance (from low to high) among assays, with only 33% of candidate donors classified identically by five different kits [27]. In the absence of a serological gold standard, our results and previous data [27] support the need for a confirmatory second serological test following a positive screening result. Only confirmed seropositive subjects should be deferred from donation. The use of a highly sensitive screening test followed by a highly specific confirmatory test would increase overall testing accuracy and allow the correct identification of donors with the potential to transmit the disease. We propose that this two-step strategy should be systematically implemented in donor screening programs. For inter-laboratory comparability, national guidelines and legislation should specify the serological tests to be used for both screening and confirmation. Both screening and confirmatory assays should demonstrate good repeatability, and, ideally, they should target different antigens.

In the present study, LAMP testing for *Plasmodium* DNA was performed in a small subset of seropositive subjects and resulted in negative results in all instances. A study recently conducted in Spain involved a larger number of samples and yielded identical results [32]. We propose that confirmed seropositive subjects should be further tested with molecular methods to exclude ongoing infection.

Seropositivity for anti-*Plasmodium* antibodies was higher among donors of sub-Saharan African origin (42.9%) compared to Europeans (3.4%). Consequently, a larger proportion of individuals from malaria-endemic regions are deferred, leading to the loss of donations from donors with rare blood groups (e.g., Duffy-negative) [33]. Among donors who initially tested positive and underwent repeat testing, 66% resulted seronegative upon retesting. The proportion of discordant results increased with increasing time interval between tests (49% after 1 month, 77–79% after 1–3 years, and 94% after >3 years), suggesting that these discrepancies are mainly due to seroreversion, although limited test repeatability cannot be excluded. A seroreversion rate of 77% after 1 year may support the idea to reduce the deferral period for seropositive donors from three years to one. The use of a two-step serological testing together with the definition of a shorter deferral period would maintain blood safety while preserving donations from individuals originating from endemic regions, thereby improving the availability of rare blood types.

Seropositivity for *T. cruzi* antibodies was 0.7%, markedly lower than that observed for malaria. None of the seropositive individuals met criteria for chronic CD diagnosis according to current WHO guidelines [29]. A national survey recently conducted in Italy [11] reported an even lower prevalence of 0.1%. The difference between these values may partly reflect differences in study duration and sampling periods (2020–2021 vs. 2016–2024). In the national survey, approximately 50% of individuals testing positive on screening were confirmed positive by the confirmatory assay and consequently diagnosed with chronic CD.

## 5. Conclusions

Since early 2016, the Laboratory of Parasitology at Pisa University Hospital has performed serological screening of candidate blood donors residing in northwestern Tuscany who were identified by blood centres as being at risk of transmitting malaria and/or CD based on a standardized risk-factor questionnaire. Over an eight-year period, the seroprevalence of anti-*Plasmodium* antibodies was 6.8%, resulting in the temporary deferral of 238 out of 3754 donors for three years. In the same period, the seroprevalence of anti-*T. cruzi* antibodies was 0.7%, leading to the permanent deferral of 32 out of 4285 donors.

Malaria seropositivity, as expected, was higher among donors originating from malaria-endemic regions, particularly sub-Saharan Africa, and varied according to the commercial ELISA kit employed. A considerable proportion of initially seropositive individuals tested negative upon repeat testing, with seroreversion increasing over time and reaching 77% after one year. These findings suggest that malaria seropositivity should be confirmed with a second independent assay and that confirmed seropositive donors could be deferred for one year rather than three. Such an approach would maintain transfusion safety while minimizing the loss of donations, particularly from donors carrying rare blood groups, and we therefore propose its adoption, together with the indication of the recommended tests, by donor screening programs.

Finally, the implementation of a national seroprevalence survey for malaria and CD is warranted to strengthen the available evidence and inform potential revisions of national blood donation guidelines as well as legislation regarding the prevention of transfusion-transmitted infections.

## Figures and Tables

**Table 1:** Anti-*Plasmodium* IgG seropositivity. The table shows the total number of donors, the number (N) of donors with positive and negative results of anti-*Plasmodium* antibody testing, and the resulting seropositivity (%) with its 95% Confidence Interval (CI), according to sex, age group, and WHO region of origin of the donor, as well as according to ELISA and year of testing. Results for 2024 are partial, as they include only the first 2 months of the year.

Donor or Test Characteristics		Donors at Risk (N)	Seronegative Donors (N)	Seropositive Donors (N)	Seropositivity (%)	95% CI
Sex	Female	1492	1408	84	5.6	4.6–6.9
	Male	2262	2108	154	6.8	5.8–7.9
Age group (years)	18–29	849	788	61	8.1	6.5–10.2
	30–39	1064	1006	58	6.0	4.7–7.6
	40–49	911	847	64	7.0	5.6–8.9
	50–70	823	779	44	5.7	4.3–7.5
	na	107	96	11	10.8	6.2–18.1
WHO region	AFR	173	105	68	42.9	36.1–49.9
	AMR	321	294	27	8.6	6.0–12.2
	EMR	62	61	1	3.0	0.8–11.4
	EUR	2705	2614	91	3.4	2.8–4.2
	SEAR	159	142	17	10.6	6.7–16.4
	WPR	48	44	4	8.3	3.1–20.4
	na	286	256	30	11.0	7.9–15.1
Assay	DRG	2270	2095	175	8.2	7.1–9.3
	EI	1484	1421	63	4.8	3.8–5.9
Year	2016	423	393	30	7.6	5.5–10.5
	2017	519	497	22	5.4	3.8–7.7
	2018	578	530	48	8.6	6.6–11.2
	2019	654	585	69	10.4	8.3–12.9
	2020	543	527	16	3.6	2.4–5.6
	2021	225	207	18	8.3	5.3–12.6
	2022	245	231	14	6.7	4.2–10.6
	2023	425	408	17	4.4	2.8–6.7
	2024	142	138	4	2.8	1.0–7.1
Total		3754	3516	238	6.8	6.1–7.7

**Table 2:** Anti-*T.cruzi* IgG seropositivity. The table shows the total number of donors and the number (N) of donors with positive and negative results of anti-*T. cruzi* antibody testing, and the resulting seropositivity (%) with its 95% Confidence Interval (CI), according to sex, age group, and WHO region of origin of the donor, as well as according to ELISA and year of testing. Results for 2024 are partial, as they include only the first 2 months of the year.

Donor or Test Characteristics		Donors at Risk (N)	Seronegative Donors (N)	Seropositive Donors (N)	Seropositivity (%)	95% CI
Sex	Female	1785	1770	15	0.8	0.5–1.4
	Male	2500	2483	17	0.7	0.4–1.1
Age group (years)	18–29	856	849	7	0.8	0.4–1.7
	30–39	1042	1037	5	0.5	0.2–1.1
	40–49	1252	1240	12	1.0	0.5–1.7
	50–70	1093	1085	8	0.7	0.4–1.5
	na	42	42	0	0.0	-
WHO region	AFR	19	19	0	0.0	-
	AMR	544	539	5	0.9	0.4–2.2
	EMR	8	8	0	0.0	-
	EUR	3413	3386	27	0.8	0.5–1.2
	SEAR	9	9	0	0.0	-
	WPR	6	6	0	0.0	-
	na	286	286	0	0.0	-
Assay	ICT	3160	3134	26	0.8	0.6–1.2
	ELISA	1125	1119	6	0.5	0.2–1.2
Year	2016	992	988	4	0.4	0.2–1.1
	2017	519	515	4	0.8	0.32.0
	2018	497	493	4	0.8	0.3–2.1
	2019	549	546	3	0.5	0.2–1.7
	2020	493	484	9	1.8	1.0–3.5
	2021	466	461	5	1.1	0.4–2.6
	2022	280	278	2	0.7	0.2–2.8
	2023	383	382	1	0.3	0.01.8
	2024	106	106	0	0.0	-
Total		4285	4253	32	0.7	0.5–1.1

## Data Availability

The data presented in this study are available upon reasonable request to the corresponding author.
